# Design of Miniaturized Dual-Band Microstrip Antenna for WLAN Application

**DOI:** 10.3390/s16070983

**Published:** 2016-06-27

**Authors:** Jiachen Yang, Huanling Wang, Zhihan Lv, Huihui Wang

**Affiliations:** 1 School of Electronic Information Engineering, Tianjin University, 92 Weijin Road, Tianjin 300072, China; yangjiachen@tju.edu.cn; 2 Institute of Advanced Technology, Chinese Academy of Sciences, 1068 Xueyuan Avenue, Shenzhen University Town, Shenzhen 518055, China; lvzhihan@gmail.com; 3 Department of Engineering Jacksonville University 2800 University Blvd N, Jacksonville, FL 32211, USA; hwang1@ju.edu

**Keywords:** WLAN, microstrip antenna, dual-frequency, HFSS

## Abstract

Wireless local area network (WLAN) is a technology that combines computer network with wireless communication technology. The 2.4 GHz and 5 GHz frequency bands in the Industrial Scientific Medical (ISM) band can be used in the WLAN environment. Because of the development of wireless communication technology and the use of the frequency bands without the need for authorization, the application of WLAN is becoming more and more extensive. As the key part of the WLAN system, the antenna must also be adapted to the development of WLAN communication technology. This paper designs two new dual-frequency microstrip antennas with the use of electromagnetic simulation software—High Frequency Structure Simulator (HFSS). The two antennas adopt ordinary FR4 material as a dielectric substrate, with the advantages of low cost and small size. The first antenna adopts microstrip line feeding, and the antenna radiation patch is composed of a folded T-shaped radiating dipole which reduces the antenna size, and two symmetrical rectangular patches located on both sides of the T-shaped radiating patch. The second antenna is a microstrip patch antenna fed by coaxial line, and the size of the antenna is diminished by opening a stepped groove on the two edges of the patch and a folded slot inside the patch. Simulation experiments prove that the two designed antennas have a higher gain and a favourable transmission characteristic in the working frequency range, which is in accordance with the requirements of WLAN communication.

## 1. Introduction

In recent years, with the rapid development of wireless communication technology, the WLAN communication system has also flourished, and the applied range in the market is increasingly wide [1,2]. WLAN communication systems generally require two-way sending and receiving data in a fast, high-efficiency and reliable way, which is reflected in the antenna subsystem. The antenna is an important part of the wireless communication system [3,4]. Modern society has entered into the information age, and people present higher requirements for the antenna, that is, the antenna not only has a wider frequency band, smaller size and is easier to install, but also has a high radiation efficiency and anti-interference performance, and other characteristics [5]. Therefore, the study of multiband and miniaturized antennas becomes an important issue in the field of antennas [6]. Compared with the traditional microwave antenna, the microstrip antennas are low profile, small size, low cost and light weight, which can meet the demands of miniaturization. However, microstrip antennas inherently have narrow bandwidth; hence, the study of dual-band microstrip antennas is necessary.

**Figure 1:**
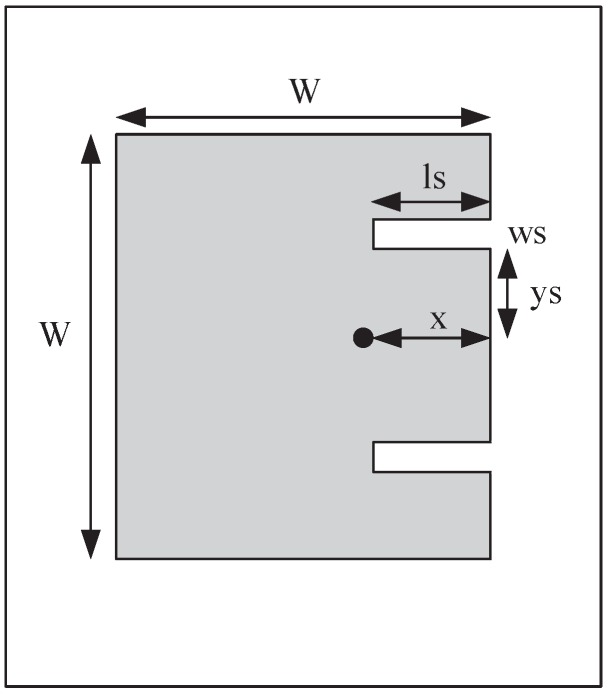
Structure of a microstrip antenna.

At present, extensive studies of dual-band microstrip antennas applied in WLAN have been carried out, and a lot antenna types which work in a dual-band have been put forward, such as Dipole antennas [7], Planar Inverted-F antennas [8], Planar Monopole antennas [9] and Quasi-Yagi antennas [10,11]. These antennas are simple in structure and low in production cost, which are suitable for the use of WLAN devices. The research of microstrip antennas is mainly focused on small scale, broadband, multi polarization, multi band and high gain [12,13,14], etc. For example, Heng-Tung Hsu et al. [15] have designed a microstrip antenna for dual band operation, as shown in Figure 1.

This paper mainly studies the dual frequency characteristics of microstrip antennas. Two dual frequency microstrip antennas designed with HFSS, that is manufactured by Ansoft of the United States, can be applied to WLAN. The simulation results indicate that the antenna has a satisfactory performance.

The rest of the paper is organized as follows. In Section 2, the paper introduces the related principle and method of microstrip antenna design. A broadband dual band printed antenna for WLAN is designed in Section 3. In Section 4, a dual frequency dual band microstrip antenna is designed, which is used to realize the dual band operation on the radiation side. Section 5 is the summary of the paper.

## 2. Design Theory

### 2.1. Introduction to Microstrip Antennas

A microstrip antenna is a resonant radiator, whose radiation field is produced by the electromagnetic field of mutual-motivation between the upper radiation patch edges of microstrip antenna and the grounding plate, and constantly radiates out electromagnetic waves through the gap between them [16,17]. The parameters of the antenna are the measure of the quality of the antenna. The microstrip antenna designed in this paper will mainly study the bandwidth and the gain. The bandwidth is the frequency range of the antenna when the antenna is off the center of the operating frequency; meanwhile, the antenna’s performance parameter is reduced to the allowable value [18,19]. Generally, the bandwidth of the microstrip antenna is in 0.7%–7%. The gain of the antenna is the ratio of the radiation power intensity generated by the actual designed antenna to the ideal radiation unit at the same point in space as the same input power is equal.

### 2.2. The Feeding of the Antenna, Miniaturization and Dual-Band Technology

**Figure 2:**
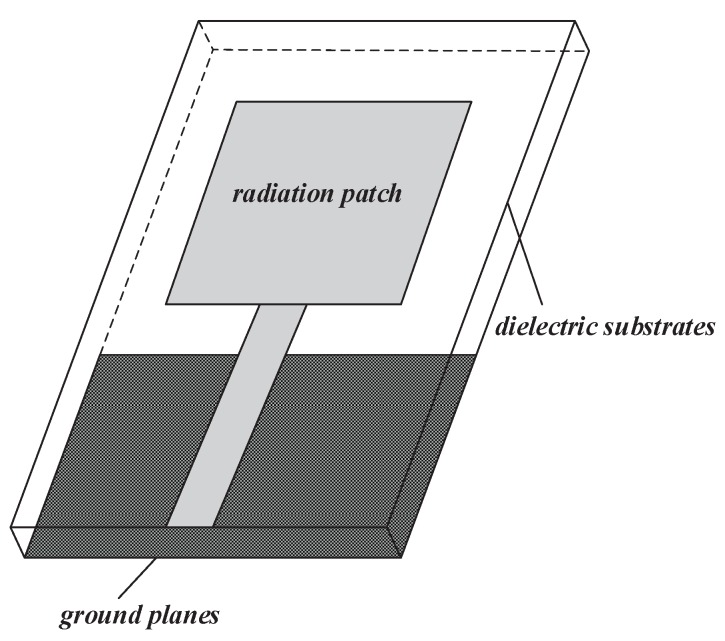
Structure chart of a printed monopole antenna.

The typical structure of printed monopole antenna is shown in Figure 2. It has advantages of small size, light weight, easy to integrate and mass production [20,21,22]. The feed means of the antenna is microstrip line feeding. The manufacturing process of microstrip line is simple, easy to integrate with other active and passive circuit components, which is conducive to realizing the miniaturization of the circuit system and improving the degree of integration.

When using coaxial feed, the probe used for feeding is stretched into the resonant cavity to motivate the patch antenna. The advantages of the coaxial feed is that the feeding points can be selected at any desired position of the patch, and the coaxial feeder located in below the ground plane and the patch antenna located in the ground floor avoid the effect of the feeder on the antenna radiation.

The bandwidth and gain of the antenna are closely related to the structure and size of the antenna. Reducing the size of the antenna will reduce the efficiency of the antenna and narrow the bandwidth. In many cases, especially in aerospace satellite communication and mobile communication, due to the limitation of physical space, small antennas are urgently needed [23,24]. Therefore, it is necessary to reduce the size of the antenna while improving the performance of the antenna.

With the development of WLAN technology and other communication technologies, the demand for dual-frequency and multi-frequency antennas is increasing. In light of this situation, the multi-frequency operation method of the microstrip antenna has been studied extensively. There are two kinds of basic methods: monolithic chip method and multi chip method. Monolithic chip method can be divided into monolithic multi mode method and monolithic loading method. Monolithic multi mode method works in different modes of patch simultaneously, while monolithic loading method adopts loading to form different resonance frequencies. For monolithic microstrip patch antenna, in addition to the use of multi mode method, the load method can also be employed to achieve multi-frequency work. In addition, low-frequency ratio of the two working frequencies can be obtained by using the loading method [25]. Commonly used forms of loading slots include rectangular slots, U-shaped grooves and L-shaped grooves. Through the adjustment of the length and width of the gap, the antenna can achieve excellent matching characteristics and multi-frequency characteristics [26,27].

## 3. Design of Miniaturized Dual-Band Broadband Printed Antennas

This part designs a broadband dual-frequency printed monopole antenna for WLAN, which works in two frequency bands of 2.4 GHz–2.52 GHz and 4.5 GHz–7.5 GHz, covering 2.4 GHz, 5.2 GHz and 5.8 GHz, three working frequencies of WLAN. The gain of 4.4 dB, 4.1 dB and 5 dB are obtained in the maximum radiation direction of the three frequency bands, respectively.

### 3.1. Antenna Structure Design

**Figure 3:**
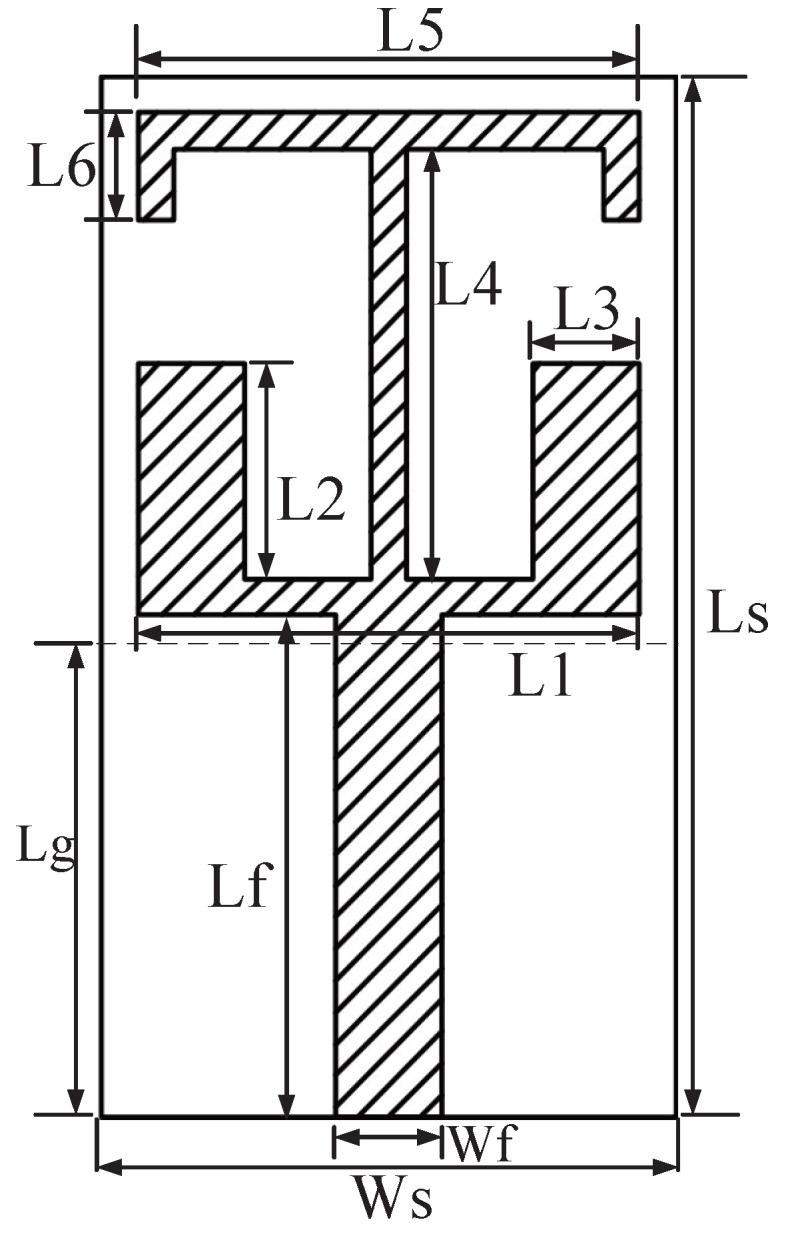
Antenna structure chart.

In Figure 3, the structure chart of printed monopole antenna is presented. The antenna is printed on the FR4 antenna substrate with a relative dielectric constant of 4.4. The size of the substrate is Ws × Ls, the thickness is 1.6 mm. The ground plane of the antenna is printed on the back ground of substrate, whose length and width are represented by Lg and Ws, respectively. In addition, the main part of the antenna is printed on the front of the substrate. The antenna is fed by a microstrip line with a characteristic impedance of 50 Ω, and the size of microstrip line is Wf × Lf. The radiating element of the antenna is composed of two rectangular patches and a T-shaped folded patch. The length of the rectangular patch is L2, and the width is L3. The length of the vertical portion of the T-shaped is L4, the length of horizontal section is L5, and the folding part is L6.

### 3.2. The Structural Analysis of the Antenna

According to the working principle of the monopole antenna, using the principle of mirror image, the ground plane can be introduced to reduce the length of the half-wave dipole antenna by half, which is equivalent to 1/4 wavelength of the monopole antenna. Then, the resonant frequency of printed monopole antenna is expressed as:

(1) $$f=\frac{c}{4L\sqrt{\varepsilon_{r}}}$$

where *c* is the speed of light, *L* is the resonant path length of the radiator, and $\varepsilon_{r}$ is the relative dielectric constant of the dielectric-slab. The antenna adopts a 50 Ω microstrip to feed, and the width of the microstrip feeder can be obtained by the following equation:

When *w*/*h* > 1,

(2) $$Z_{0}=Z_{f}2\pi \sqrt{\varepsilon_{eff}}\ln\left(8\frac{h}{w}+\frac{w}{4h}\right)$$

(3) $$\varepsilon_{eff}=\frac{\varepsilon_{r}+1}{2}+\frac{\varepsilon_{r}-1}{2}\left[{\left(1+12\frac{h}{w}\right)}^{\frac{-1}{2}}+0.04{\left(1-\frac{w}{h}\right)}^{2}\right]$$

When *w*/*h* < 1,

(4) $$Z_{0}=\frac{Z_{f}}{\sqrt{\varepsilon_{eff}}\left(1.393+\frac{w}{h}+\frac{2}{3}ln\left(\frac{w}{h}+1.444\right)\right)}$$

(5) $$\varepsilon_{eff}=\frac{\varepsilon_{r}+1}{2}+\frac{\varepsilon_{r}-1}{2}{\left(1+12\frac{h}{w}\right)}^{\frac{-1}{2}}$$

In the formula, $Z_{f}$ = 376.8 Ω represents free space wave impedance, *h* represents the height of medium plate, *w* represents the width of microstrip line, and $\varepsilon_{eff}$ represents the effective dielectric constant of the dielectric plate.

We run the commercial software Ansoft HFSS to simulate the structure of the antenna. The effects of various parameters on the performance of the antenna are studied in the following.

**Figure 4:**
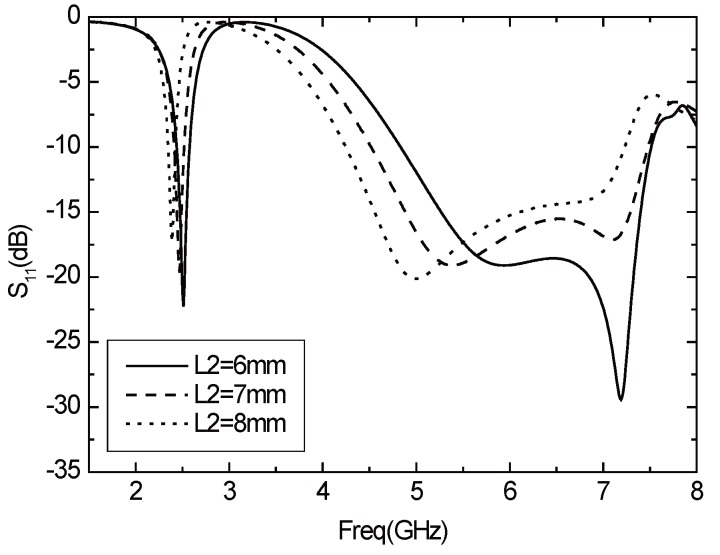
Parameter *S*${}_{11}$ varies with L2.

Changing the range of L2, which denotes the unit length of rectangular radiation, and keeping other parameters unchanged, the changes of return loss *S*${}_{11}$ with L2 are shown in Figure 4. It can be seen from the figure that L2 mainly affects the bandwidth and the resonant point at a high band. As L2 becomes larger, the resonant frequency of 2.4 GHz band moves to the left, the resonant frequency at high frequency significantly decreases, and the matching situation becomes worse.

**Figure 5:**
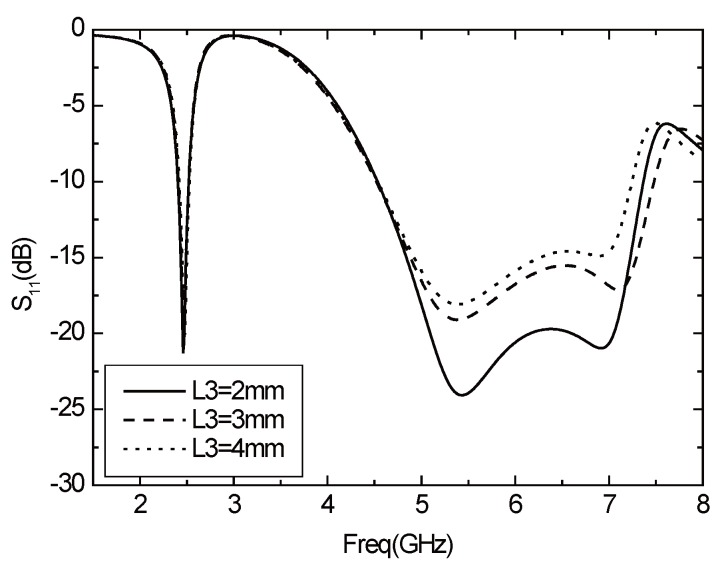
Parameter *S*${}_{11}$ varies with L3.

The width of the rectangular radiating element is L3. Figure 5 illustrates the results of *S*${}_{11}$ parameter varies with L3. L3 will not affect the resonant frequency of two frequency bands but will affect the matching of the antenna. As L3 becomes larger, the matching situation becomes worse, and the bandwidth is also reduced.

**Figure 6:**
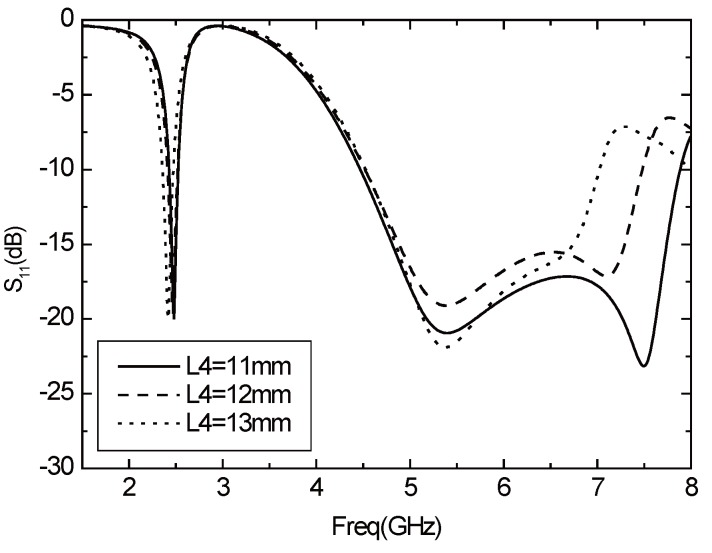
Parameter *S*${}_{11}$ varies with L4.

The vertical part of the T-sharped element is L4, and the *S*${}_{11}$ varies with L4 as shown in Figure 6. The resonant frequency of 2.4 GHz band decreases with the increase of L4, and the resonant frequency of the high frequency band does not change with the L4. While the L4 becomes larger, the bandwidth of the high frequency band is obviously reduced.

**Figure 7:**
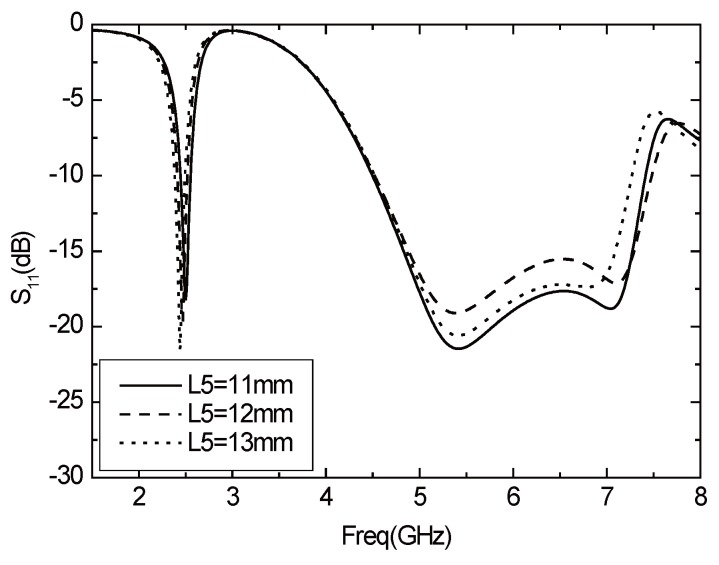
Parameter *S*${}_{11}$ varies with L5.

The length of the horizontal part of the T-sharped element is L5. The value of L5 is changed, and the other parameters are constant, while the changes of *S*${}_{11}$ with L5 are shown in Figure 7. As shown in the chart, the resonance frequency of the low frequency band decreases with the increasing of L5, and the resonant frequency of the high frequency band does not vary with L5.

**Figure 8:**
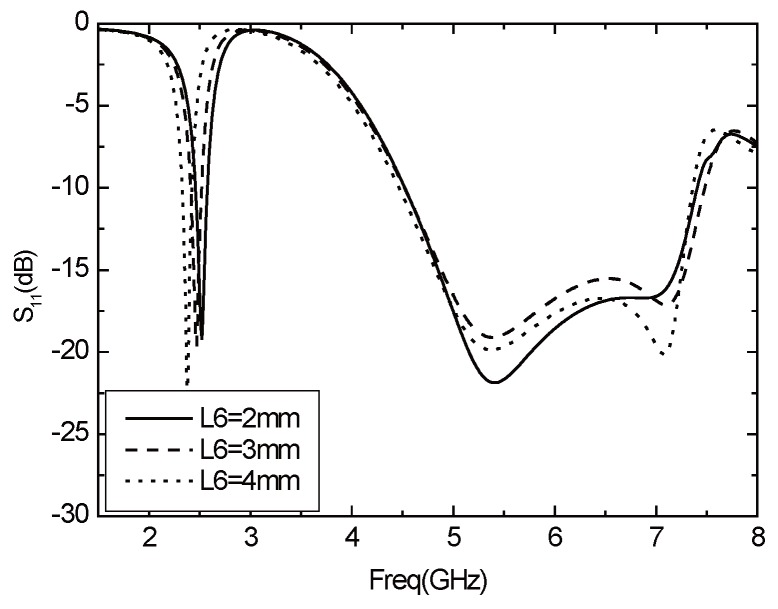
Parameter *S*${}_{11}$ varies with L6.

The folding part of the T-sharped unit is L6, and the changes of *S*${}_{11}$ with L6 are shown in Figure 8. As shown in the chart, the resonant frequency of the low frequency band decreases with the increasing of L6, and the resonance frequency of the high frequency band does not vary with L6.

### 3.3. The Results of Antenna Simulation

After optimization, the final parameters of the antenna are shown in Table 1. The overall size of the antenna is 16 mm × 29 mm, the structure is compact, and the horizontal direction of the T-shaped element is folded down to reduce the antenna size. The miniaturization of the antenna can be applied to the wireless network card and other small devices.

**Figure 9:**
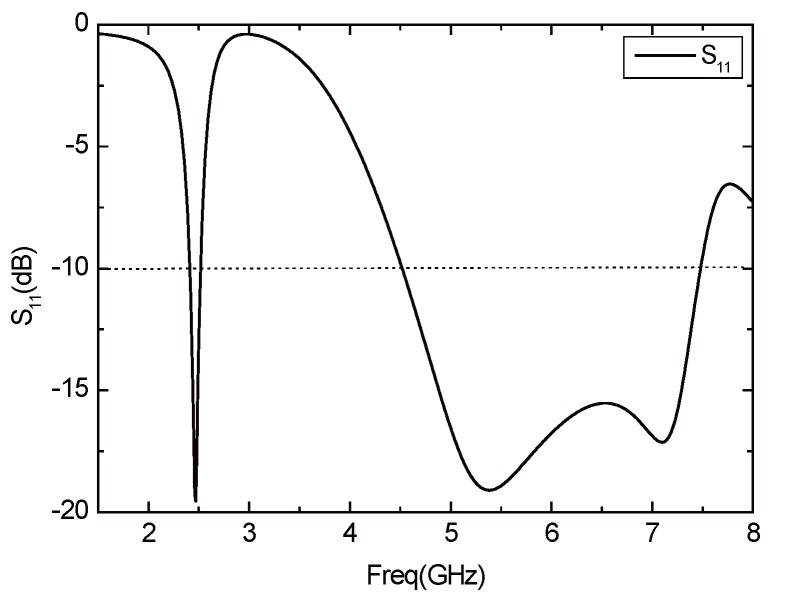
Parameter *S*${}_{11}$ of the antenna.

**Figure 10:**
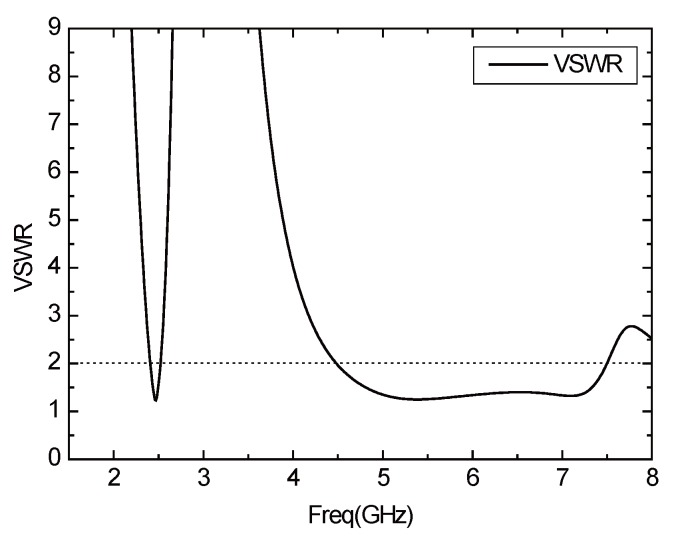
VSWR curve of the antenna.

**Figure 11:**
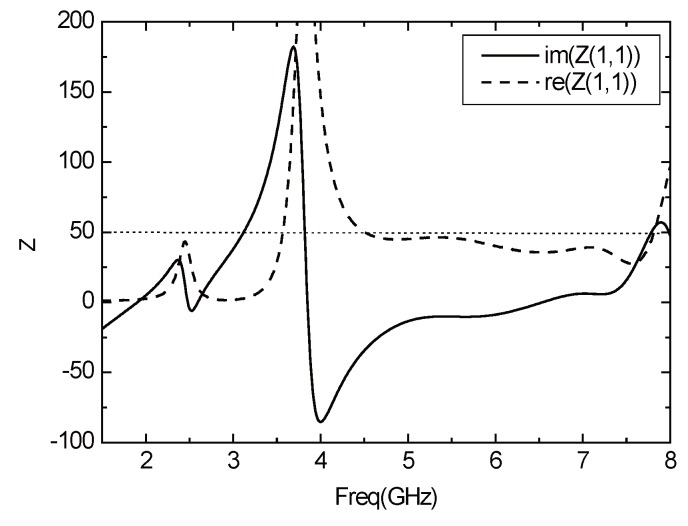
Input impedance curve of the antenna.

According to the optimized parameters of the antenna, the *S*${}_{11}$ parameter curve of the antenna is shown in Figure 9. The antenna works in the two frequency bands of 4.5 GHz–7.5 GHz and 2.4 GHz–2.52 GHz, the impedance bandwidth reaches 3 GHz and 120 MHz, respectively, covering the 2.4 GHz, 5.2 GHz and 5.8 GHz three operating frequency bands of WLAN. In addition, the working broadband is realized. Figure 10 shows the Voltage Standing Wave Ratio (VSER) of antenna, and it can be inferred that the values of the standing wave ratio are less than two and the antenna has good transmission characteristics. Figure 11 depicts the input impedance of the antenna. The input impedance in the passband is about 50 Ω, and the antenna can be well matched with the microstrip feeder.

**Figure 12:**
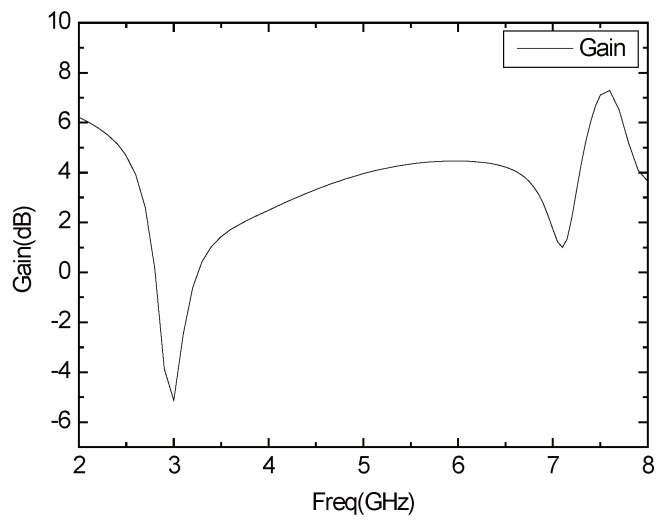
The gain curve of the antenna.

**Figure 13:**
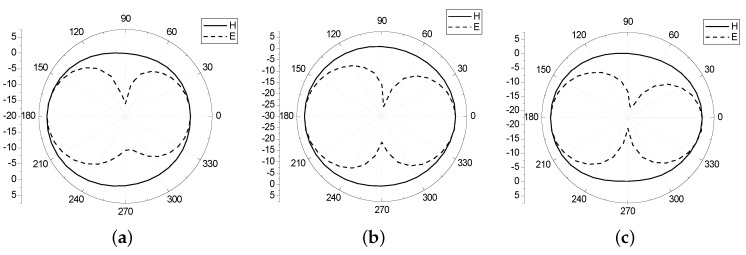
Antenna pattern (**a**) 2.45 GHz; (**b**) 5.2 GHz; (**c**) 5.8 GHz.

Figure 12 shows the gain of the antenna. As shown in the chart, the gain of the antenna in the 2.4–2.5 GHz band can reach 4–5 dB, and the gain in the 5 GHz band can reach more than 4 dB. The antenna realizes the miniaturization and achieves a higher gain. Figure 13 illustrates the E plane and H plane antenna patterns in the 2.45 GHz, 5.2 GHz and 5.8 GHz. The E plane graph is “*∞*” shaped, and the H plane graph is circular, which indicates that the antenna has outstanding omnidirectional characteristics.

## 4. Design of Dual-Band Microstrip Antenna

In this section, the design of the rectangular dual-band microstrip antenna for WLAN is studied. The influence of different slot styles on the antenna performance is discussed. A microstrip antenna with a stepped groove is designed and improved, and the dual-band operation is realized by opening a pair of symmetrically folded grooves. The antenna works in two frequency bands of 2.4 GHz–2.46 GHz and 5.16 GHz–5.4 GHz, and the antenna gain can achieve 4 dB in most of the working frequencies. The antenna patch is a square patch whose length of a side is 25 mm, which ensures the appropriate gain of the antenna while reducing the volume of the antenna.

### 4.1. Antenna Structure Design

**Figure 14:**
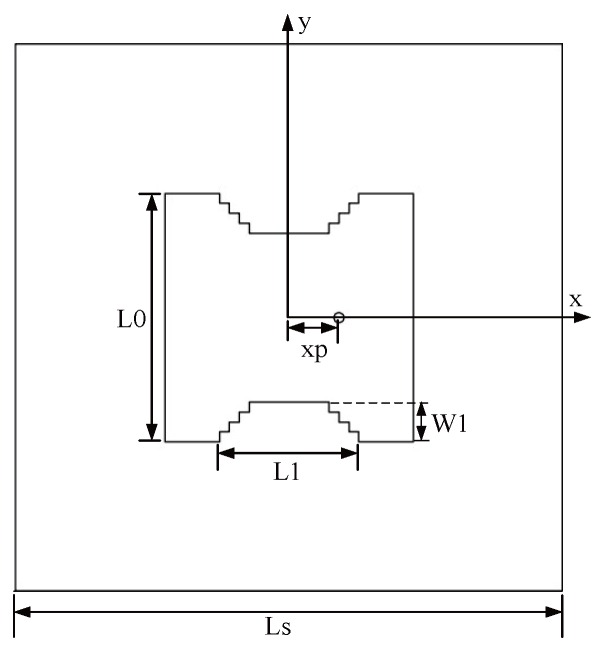
Antenna structure.

The antenna structure is shown in Figure 14. The antenna is printed on a square FR4 substrate, the thickness and length of which are 1.6 mm and Ls, respectively. The radiating element of the antenna is a square patch, whose size length is L0. The mode of feeding adopts the coaxial feeding of characteristic impedance 50 Ω, and the distance from the feed point to the center of the patch is xp. In addition, the trap cut slot is opened on the opposite sides of the antenna, the length and width of each step are all 1 mm, the total length of the ladder shaped groove is L1, and the total width is W1. We simulate the design of the antenna with the simulation software HFSS.

### 4.2. The Choice of Antenna Slotting Form

The microstrip antenna can be slotted to achieve multi band, and the slot form is varied. The key of the design is to adopt the appropriate slotting form. The effects of different slot forms on the performance of the antenna are discussed in the following.

**Figure 15:**
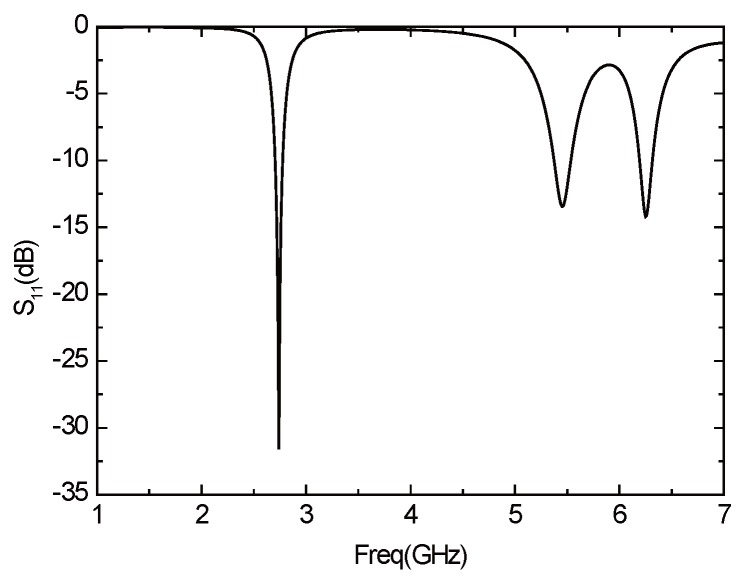
Parameter *S*${}_{11}$ of the antenna without a slot.

The initial parameters of the antenna are unchanged, and the antenna is not slotted, the return loss of the antenna is shown in Figure 15. The antenna has three resonant frequencies—2.74 GHz, 5.46 GHz, and 6.25 GHz, which are all not in the desired frequency band. Therefore, the structure of the antenna must be improved.

**Figure 16:**
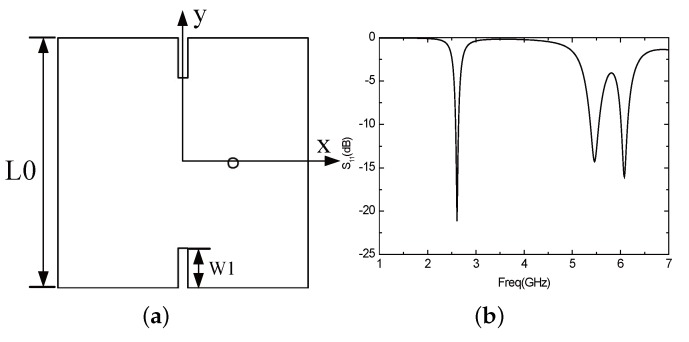
(**a**) Antenna structure with a rectangular slot; (**b**) The parameter *S*${}_{11}$ of the antenna with a rectangular slot.

When the antenna is not slotted, the resonant frequency of the antenna is too high. However, the resonant frequency can be moved to the left with the method of slotting, so that the size of the antenna can be reduced. Accordingly, the two edges of the antenna are opened a narrow rectangular groove with a width of 1 mm, a length of W1, as shown in Figure 16a. Figure 16b illustrates the parameter *S*${}_{11}$ of the antenna under this circumstance. The antenna resonates in the three frequency bands of 2.6 GHz, 5.45 GHz and 6.08 GHz. It can be seen that the frequency of the antenna, to some extent, moves left, but still does not resonate in the required frequency band. Increasing W1 can further reduce the operating frequency of the antenna, but the antenna gain will also be reduced, and the directional diagram will become worse to some extent. Hence, we can consider increasing the width of the gap.

**Figure 17:**
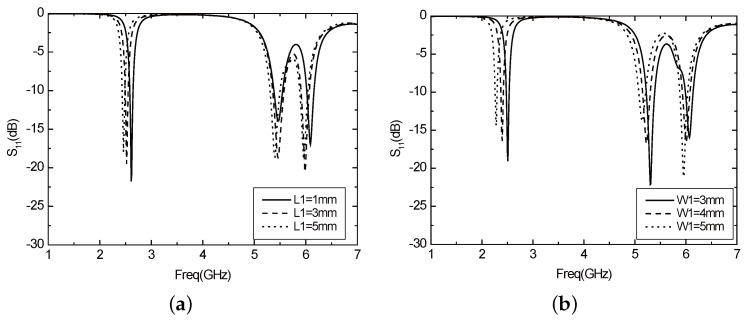
(**a**) The parameter *S*${}_{11}$ varies with L1 when opening the rectangular slot; (**b**) The parameter *S*${}_{11}$ varies with W1 when opening the rectangular slot.

**Figure 18:**
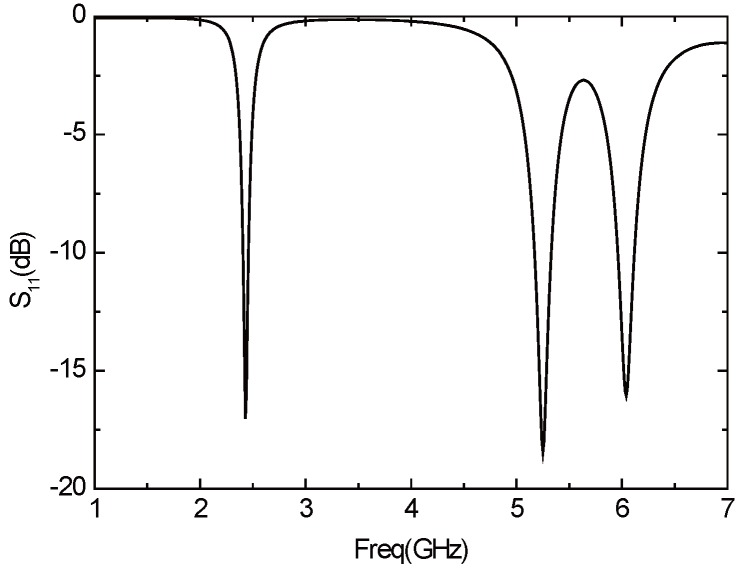
*S*${}_{11}$ parameter curve of the optimized antenna.

By adjusting the width W1 and length L1 of the slot, it can be seen from the parameter *S*${}_{11}$ of the antenna that the resonance frequency of the antenna will be reduced as the size of the antenna is increased, as shown in Figure 17. Figure 18 describes the *S*${}_{11}$ parameter curve of the optimized antenna. At this point, L1 = 9 mm, W1 = 3.7 mm, the resonance frequency of the antenna is 2.43 GHz, 5.25 GHz and 6.05 GHz, covering 2.4 GHz and 5.2 GHz operating frequency bands of WLAN. However, there is a useless resonance frequency (6.05 GHz), which will affect the normal operation. Thus, the following consideration is employing other forms to try to eliminate this effect.

**Figure 19:**
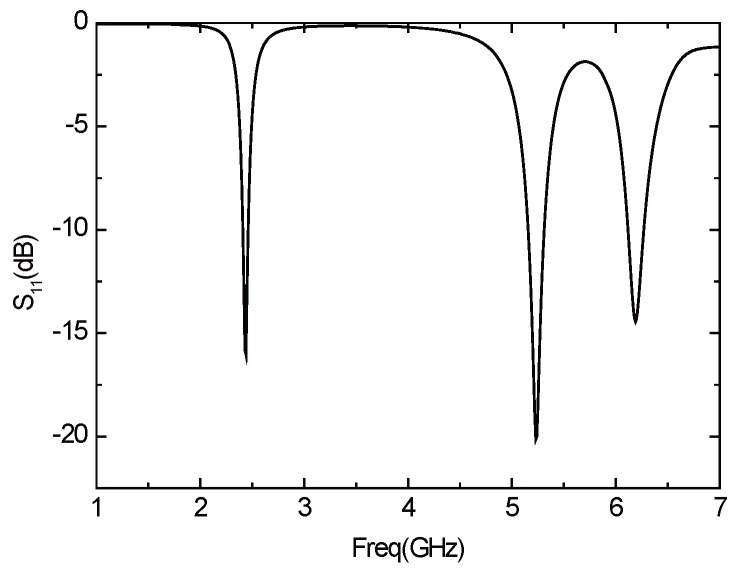
*S*${}_{11}$ parameter curve of the antenna with a stepped groove.

The rectangular slot can obtain two frequency points that are required, but meanwhile, there will be unwanted frequency points that cannot be removed, so we switch to the ladder type slot as shown in Figure 14. By properly adjusting the size of the slot, the performance of the antenna can be obtained, as shown in Figure 19. The antenna has three resonant frequencies—2.44 GHz, 5.24 GHz and 6.2 GHz. The third frequency of the ladder type is larger than that of the rectangular slot, but it is still possible to have a passive effect on the performance of the antenna.

### 4.3. Improved Dual-Band Microstrip Antenna

**Figure 20:**
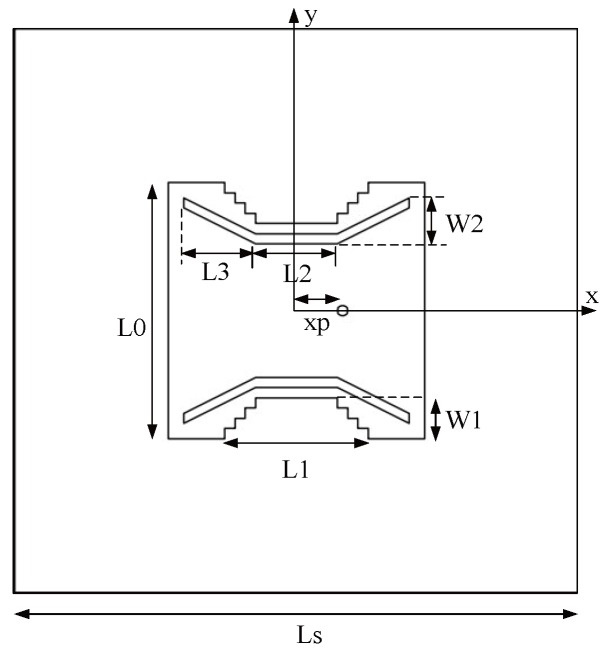
Improved antenna structure.

In order to eliminate the third frequency point, we now improve the antenna structure shown in Figure 14. We open two symmetrical folding shape slots on the basis of the above antenna, the improved antenna structure as shown in Figure 20. The length of slotted horizontal section is L2, the length of collapsible sections is L3, and the length of vertical component is W2. In Figure 20, the length of dielectric slab is Ls = 55 mm, the length of square patch of antenna is L0 = 25 mm, and the size of ladder shaped groove is L1 = 14 mm, W1 = 4 mm. The size of the folding groove is L2 = 8 mm, W2 = 4.5 mm, L3 = 7 mm, and the distance from the patch center to the feed point is xp = 4.5 mm. According to the parameters of the improved antenna, the simulation results are as follows.

**Figure 21:**
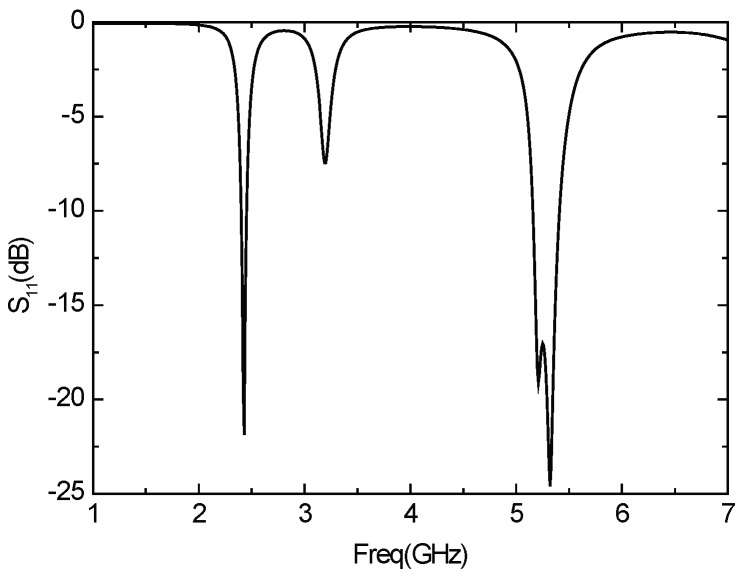
*S*${}_{11}$ parameter curve of the improved antenna.

**Figure 22:**
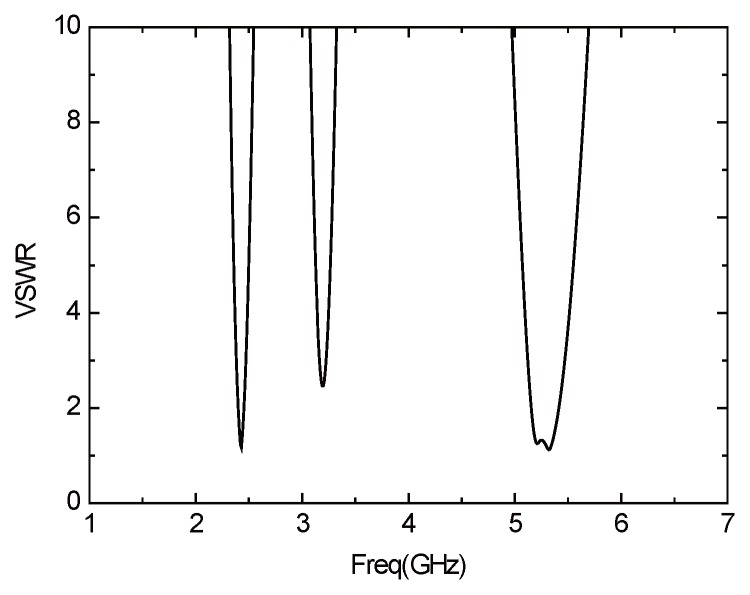
VSWR curve of the improved antenna.

**Figure 23:**
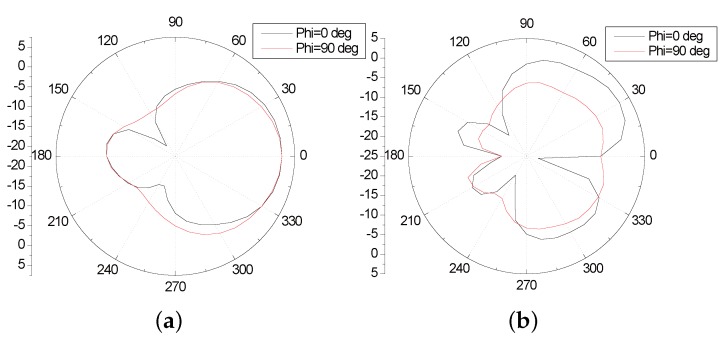
The directional pattern of the improved antenna (**a**) 2.43 GHz; (**b**) 5.2 GHz.

**Figure 24:**
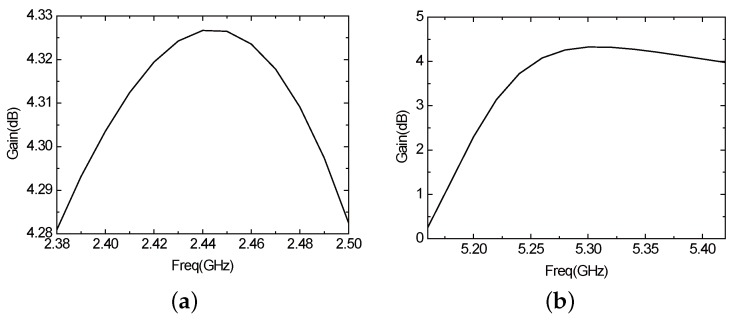
The gain of the improved antenna (**a**) 2.43 GHz; (**b**) 5.2 GHz.

Figure 21 presents the *S*${}_{11}$ parameter curve of the improved antenna. The antenna consists of two resonant frequencies—2.43 GHz (2.4 GHz–2.46 GHz) and 5.2 GHz (5.16 GHz–5.4 GHz). The bandwidth, that when the return loss of the antenna is less than −10 dB, are 60 MHz (2.4 GHz–2.46 GHz) and 240 MHz (5.16 GHz–5.4 GHz), which can basically meet the demand of WLAN in the two frequency bands. Figure 22 is the curve of voltage standing wave ratio (VSWR) of the improved antenna. In the two frequency bands above, the VSWR is less than two, which indicates there exists positive transmission characteristics. Figure 23 illustrates the directional pattern of the antenna at the far field. It can be seen that the directional pattern of the antenna is better in the 2.43 GHz, but it becomes worse in the 5.2 GHz. Figure 24 is the gain of the antenna. The gain is greater than zero in the working band of the antenna, and the gain can achieve 4 dB in most of the frequency bands. In addition, the size of the antenna is 25 × 25 mm, which realizes the miniaturization of the antenna, and ensures the antenna gain, so that it has excellent performance.

## 5. Conclusions

In this paper, two dual-band antennas according to the demand of WLAN for multi frequency communication are designed. One of them is a dual-band printed monopole antenna, which operates in two frequency bands of 2.4 GHz–2.52 GHz and 4.5 GHz–7.5 GHz, and the impedance bandwidth reaches 120 MHz and 3 GHz, respectively, covering 2.4 GHz, 5.2 GHz and 5.8 GHz—three working frequency bands of WLAN. In addition, the gain of the antenna is greater than 4 dB, and the volume of this antenna is only 16 × 29 × 1.6 mm${}^{3}$, implementing the miniaturization of the antenna. The second antenna is a dual-band microstrip antenna, which works in two frequency bands of 2.4 GHz–2.46 GHz and 5.16 GHz–5.4 GHz, and realizes the miniaturization and dual-band operation by adopting the slotting technology. The two designed antennas have a higher gain and a favourable transmission characteristic in the operating band, which is in accordance with the requirements of WLAN communication under more complicated conditions.

## Figures and Tables

**Table 1:** The parameters of the optimized antenna (unit: mm). (the bold words in the first and the third row represent the parameters, the second and the fourth row represent the value of the corresponding parameters).

Ws	Ls	Lf	Wf	Lg	L1
16	29	14	3	13	14
L2	L3	L4	L5	L6	H
7	3	12	12	3	1.6

## References

[1] Park H., Ghovanloo M. (2014). Wireless Communication of Intraoral Devices and Its Optimal Frequency Selection. IEEE Trans. Microw. Theory Tech..

[2] Ngan T.L., Wong E.T.H., Ng K.L.S., Jeor P.K.S., Lo G.G. (2015). The Enhanced Workflow and Efficiency of the Wireless Local Area Network (WLAN)-Based Direct Digital Radiography (DDR) Portable Radiography. J. Digit. Imaging.

[3] Joseph W., Pareit D., Vermeeren G., Naudts D., Verloock L., Martens L., Moerman I. (2013). Determination of the duty cycle of WLAN for realistic radio frequency electromagnetic field exposure assessment. Progr. Biophys. Mol. Biol..

[4] Ziegler V., Schulte B., Sabater J., Bovelli S., Kunisch J., Maulwurf K., Grass E. (2012). Broadband 57–64 GHz WLAN communication system integrated into an aircraft cabin. IEEE Trans. Microw. Theory Tech..

[5] Wang Z., Lee L.Z., Psychoudakis D., Volakis J.L. (2014). Embroidered multiband body-worn antenna for GSM/PCS/WLAN communications. IEEE Trans. Antennas Propag..

[6] Mehdipour A., Denidni T., Sebak A.R. (2014). Multi-band miniaturized antenna loaded by ZOR and CSRR metamaterial structures with monopolar radiation pattern. IEEE Trans. Antennas Propag..

[7] Khodabakhshi H., Cheldavi A. (2010). Irradiation of a six-layered spherical model of human head in the near field of a half-wave dipole antenna. IEEE Trans. Microw. Theory Tech..

[8] Islam M.S., Esselle K.P., Bull D., Pilowsky P.M. (2014). Converting a wireless biotelemetry system to an implantable system through antenna redesign. IEEE Trans. Microw. Theory Tech..

[9] Lee E., Hall P., Gardner P. Novel Compact Wideband or Multi-Band Planar Monopole Antenna. Proceedings of the IEEE Antennas and Propagation Society International Symposium.

[10] Shi J., Wu X., Chen Z N., Qing X., Lin L., Chen J., Bao Z.H. (2015). A Compact Differential Filtering Quasi-Yagi Antenna with High Frequency Selectivity and Low Cross Polarization Levels. IEEE Antennas Wirel. Propag. Lett..

[11] Lu H.D., Si L.M., Liu Y. (2012). Compact planar microstrip-fed quasi-Yagi antenna. Electron. Lett..

[12] Fujimoto T., Yoshitake Y. (2014). Stacked microstrip antenna fed by an L-probe for quadruple band operation. IET Microw. Antennas Propag..

[13] Wu P., Liu J.R., Xue Q. (2015). Wideband Excitation Technology of TE20 Mode Substrate Integrated Waveguide (SIW) and Its Applications. IEEE Trans. Microw. Theory Tech..

[14] Liu Y., Hao Y., Wang H., Li K., Gong S. (2015). Low RCS Microstrip Patch Antenna Using Frequency Selective Surface and Microstrip Resonator. IEEE Antennas Wirel. Propag. Lett..

[15] Hsu H., Kuo F., Lu P. (2010). Design of Wifi/WiMAX dual-band E-shaped patch antennas through cavity model approach. Microw. Opt. Technol. Lett..

[16] Ali M.T., Dzulkefli N., Abdullah R., Omar S. Design and analysis of microstrip Yagi antenna for Wi-Fi application. Proceedings of the 2012 IEEE Asia-Pacific Conference on Applied Electromagnetics.

[17] Pereira J.P.P., da Silva J.P., de Andrade H.D. (2015). A new design and analysis of a hexagonal PBG microstrip antenna. Microw. Opt. Technol. Lett..

[18] Parmanand S., Swastik G. Bandwidth and gain enhanceent in microstrip antenna array for 8 GHz frequency applications. Proceedings of the 2014 Students Conference on Engineering and Systems.

[19] Yang Z.X., Yang H.C., Hong J.S., Li Y. (2014). Bandwidth enhancement of a polarization-reconfigurable patch antenna with stair-slots on the ground. IEEE Antennas Wirel. Propag. Lett..

[20] Souza R.D., Gupta R.K. Printed dual band WLAN antenna. Proceedings of the IEEE International Conference Electro/Information Technology.

[21] Peng C.M., Chen I.F. (2011). Modeling printed monopole antenna with coplanar ground-plane by gaussian filter model analysis. Int. J. Appl. Electromagn. Mech..

[22] Yu Y., Hui H.T. (2011). Design of a mutual coupling compensation network for a small receiving monopole array. IEEE Trans. Microw. Theory Tech..

[23] Zhu H.L., Cheung S.W., Yuk T.I. (2015). Miniaturization of patch antenna using metasurface. Microw. Opt. Technol. Lett..

[24] Sharma S., Daya K.S., Sharma S., Batoo K.M., Singh M. (2015). Sol-gel auto combustion processed soft Z-type hexa nanoferrites for microwave antenna miniaturization. Ceram. Int..

[25] Kundu A., Chakraborty U., Bhattacharjee A.K. (2015). Design of compact dual-band co-axially fed microstrip antenna for 2.4/5.2/5.8 GHz WLAN applications. J. Electromagn. Waves Appl..

[26] Qing X., Chen Z.N. (2013). A wideband circularly polarized stacked slotted microstrip patch antenna. IEEE Antennas Propag. Mag..

[27] Huang C.Y., Yu E.Z. (2011). A slot-monopole antenna for dual-band WLAN applications. IEEE Antennas Wirel. Propag. Lett..

